# Using physical activity to advance a career in clinical nutrition

**DOI:** 10.1038/s41430-024-01410-2

**Published:** 2024-03-04

**Authors:** Henry C. Lukaski

**Affiliations:** 1 Retired, US Department of Agriculture, Agriculture Research Service, Grand Forks Human Nutrition Research Center, Grand Forks, ND 50202 USA; 2 Adjunct Professor, Department of Kinesiology and Public Health Education, University of North Dakota, Grand Forks, ND 58202 USA

**Keywords:** Medical research, Health care

## “If at first you don’t succeed….”

To paraphrase Drew Weissman, 2023 Co-Noble Laureate in Physiology or Medicine, the career of a scientist is not linear, but fraught with twists and turns, starts and stops with setbacks that contribute to later successes. This scenario encompasses my career in clinical nutrition: a winding path of interactions with a variety of talented people at incredible institutions. I was first introduced to nutrition science by John “Doc” Robson, head of the Department of Nutrition in the School of Public Health at the University of Michigan, and my collegiate rugby coach. Under his tutelage, I participated in field studies of the effects of various fluid and electrolyte replacement drinks, including the prototype of Gatorade, on physical performance and temperature regulation. We also used anthropometry to describe and monitor the body composition of several sport teams, which piqued my interest in the practical aspects of nutrition. Concurrent experiences in a public health clinic with Cicely Williams, a visiting professor, in tracking anthropometric changes of mothers and children participating in a supplemental food program bolstered my interest in clinical nutrition research. These formative experiences strengthened my nascent interest in exploring the interaction of diet and physical activity. The convergence of my passion for physical activity with a growing curiosity of practical nutrition motivated my change from a traditional liberal arts curriculum to the unconventional academic path combining exercise physiology, chemistry, and mathematics.
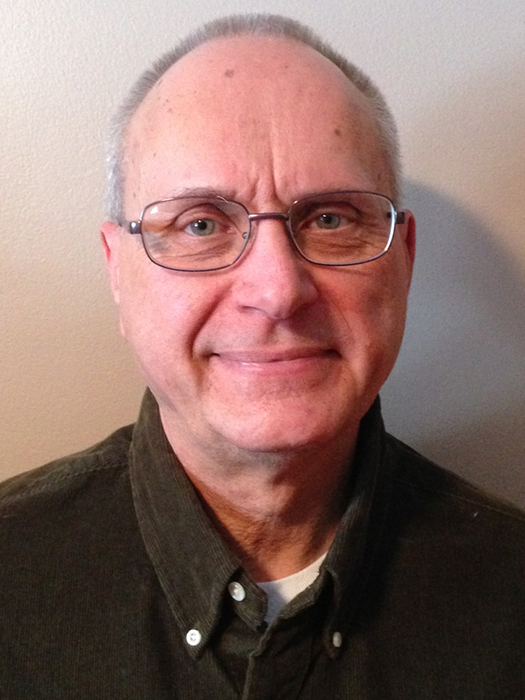


## The Pennsylvania State University experience: an encouraging start, some hurdles then progress

My pursuit of a graduate program that integrated the study of the physiology of exercise and nutritional science led me to the state-of-the-art interdisciplinary graduate program in physiology at the Pennsylvania State University. Elsworth R. Buskirk directed the new program with Jose Mendez. The Noll Laboratory for Human Performance Research was the home of this innovative program. It served as a multifunctional center for teaching and research with state-of-the-art facilities for exercise and environmental physiology research, body composition studies, and analytical laboratories. Noll Laboratory also included a kitchen and residential living areas for humans to participate in metabolic studies. Paul Baker, director of the Human Biology Program in the Department of Anthropology, generously provided me with a pre-doctoral fellowship and actively supported my pursuit of graduate training in physiology and nutrition.

My first research experiences were not without challenges. A central research theme at the Noll Laboratory was to determine the effects obesity on metabolism so my initial charge was to explore the feasibility of conducting whole-body energy metabolism studies in humans. The Department of Animal Science in the College of Agriculture had a unique resource to address this research, the Armsby Respiration Calorimeter. Although used principally to measure heat production of livestock fed different rations to determine feed efficiency, it was available for use in human studies. Preliminary trials with students demonstrated its feasibility to perform short-term studies of human energy expenditure. However, the pressing needs to remodel the chamber for human habitation, upgrade and automate oxygen and carbon dioxide gas analysis equipment, and modernize data collection systems were cost prohibitive and stalled the immediate use of the calorimeter.

My next approach was to implement a free-living method for estimation of human energy expenditure. Buskirk and Mendez introduced me to Nathan Lifson, a former colleague at the University of Minnesota, who developed the doubly labeled water (DLW) method to estimate energy expenditure and validated it in small animals [1]. Our shared enthusiasm for the novel use of DLW in studies of free-living humans with obesity weakened upon discovery that available isotope ratio mass spectrometers were unable to measure small enrichments of ^18^O thereby requiring very large amounts of ^18^O for human research. The high cost of ^18^O to support human studies of energy expenditure was extravagant and impractical, thus halting plans.

Despite these setbacks, a promising opportunity arose with the escalating clinical interest to determine muscle mass and monitor its changes in response to physiological and pathological stressors. To meet this need, Stanton Cohn, Medical Research Center at Brookhaven National Laboratory, established the novel technique of n-prompt gamma in vivo neutron activation analysis to measure total body nitrogen (TBN). Total nitrogen was a surrogate for total body protein, so it was clinically important to differentiate the muscle and non-muscle protein components of the fat-free mass (FFM). The combination of TBN values and determinations of total body potassium (TBK) from measurements of ^40^K using whole-body gamma counting enabled estimation of muscle and non-muscle protein masses based on the differential distributions of nitrogen and potassium in these components [2]. My research utilized these nuclear techniques and models and provided the first validation of the TBN-TBK model to estimate FFM in healthy men [3].

Because in vivo neutron activation analysis was a research tool, it was clear that a more practical approach was needed to estimate muscle mass. We decided to investigate the validity of 24-h urinary 3-methylhistidine (3-MeHis) excretion, a metabolite of myofibrillar protein breakdown, as an index of muscle mass. I first determined the time required to establish endogenous urinary output of 3-MeHis of physically active men fed a meat-free diet. Endogenous urinary 3-MeHis excretion was highly correlated with urinary creatinine production, the traditional biomarker of muscle mass. It was a significantly better predictor of muscle mass than TBK, TBN and FFM, and poorly related to non-muscle protein mass [4]. These findings authenticated the TBN-TBK model for estimation of protein components of the FFM.

## Pivot to physical activity and micronutrient research, and the value of team research

Employment opportunities after completion of my doctoral degree were not encouraging. Availability of academic positions in applied physiology and nutrition were sparse and unappealing. Discussions with Jose Mendez emphasized the diversity of my academic training and research experiences and mused where they might be best utilized. He reflected on his scientific work at the Institute of Nutrition for Central America and Panama in his native Guatemala and supervision of Harold Sandstead, a visiting medical student from Vanderbilt University. He contacted Sandstead, who recently was appointed the director at the United States Department of Agriculture, Agricultural Research Service (USDA-ARS), Grand Forks Human Nutrition Research Center (GFHNRC), and sought his advice on my situation.

The timing of the communication with Sandstead was serendipitous. It coincided with the availability of a post-doctoral position at the GFHNRC to respond to the needs identified in the United States Senate report to conduct research to advance scientific knowledge of the role that nutritional deficits can have on human health [5]. Importantly, this report emphasized the need to determine the public health significance of marginal intakes, in contrast to severe deficiency, thought to be prevalent in the US population, and to identify physiological and biochemical impairments associated with low intakes of nutrients. One explicit area of focus was micronutrients, specifically trace elements, that coincided with the mission of the GFHNRC. Discussions with Sandstead and Leslie Klevay highlighted their vision to develop functional tests of trace element nutriture. They also were keenly aware of the emergent public health impetus for increased physical activity in health promotion and recruited me to lead a new research thrust evaluating the interaction of trace elements and exercise on health and function.

The professional appeal of the GFHNRC was the opportunity to augment my knowledge of micronutrients with the synergy among professionals in diverse scientific fields to address the complex problem of contributing innovative data to establish dietary recommendations for trace elements. The Center had state-of-the-art facilities to carry out novel research integrating various experimental models. It had a metabolic research unit for long-term, in-patient feeding studies and an outpatient kitchen supported by dietetic staff and analytical resources. Additionally, a modern vivarium for rodent studies and a cell culture facility were available. The Center had collaborative research agreements with the University of North Dakota School of Medicine and several academic departments. These resources were fundamental in launching my career in clinical nutrition research.

I proposed the use of controlled physical stressors to identify indicators of strain (perturbations in physiological function and impaired biochemical processes) in response to the primary stressor of nutritional depletion compared to adequacy. The experimental approach was to use whole-food diets containing graded amounts of trace elements ranging from less than recommended compared to adequate intakes. My goal was to identify altered physiological function(s) that could provide novel information on nutrient-dependent biochemical functions and would complement and expand assessments using traditional nutrition assessment tools (chemical balance). I chose to apply physical challenges, consistent with activities of daily living, that would exert additional demands on trace element-dependent systems and elicit disturbances in physiological responses that were not observed under resting conditions. Following my arrival at the Center, James Penland joined me in pursuit of new functional indicators of nutritional status [6].

My initial task was to evaluate the common notion that physical training per se adversely affected trace element nutriture. In collaboration with William Bolonchuk, Department of Physical Education, we conducted an observational study and found that male athletes competing in a variety of sports and untrained men had adequate intakes of trace elements. Our key observation was the significant association between plasma magnesium concentration and aerobic capacity, an objective marker of physical fitness [7]. This finding was the first evidence of a relationship between mineral element nutriture, other than iron, and physical performance and stimulated international research interest on trace elements and physical activity.

Prospective cohort studies of the effects of physical training on trace element status followed. In collaboration with the University of North Dakota swim team and USA Swimming, we determined the interactions between chronic physical training and trace element nutritional status. Dietary iron, copper, and zinc intakes were comparable to dietary recommendations and did not change, and plasma levels were within the range of normal values after five months of training among the elite female swimmers. A key finding was that the activity of red blood cell (RBC) erythrocyte superoxide dismutase (SOD1), a copper-dependent antioxidant enzyme, increased significantly with swim training [8]. A follow-up longitudinal study of female and male swimmers, compared with non-training students, confirmed the significant increase in RBC SOD1 activity only in the swimmers after training [9]. These findings were the first evidence that, when dietary intakes were adequate, trace element nutritional status was not affected adversely by chronic, intense physical training, and that increased RBC SOD1 activity without increased copper intake was a functional adaptation of copper metabolism to physical training. These data refuted the common public belief that athletes in training should consume nutritional supplements to optimize physical performance and provided early evidence of training-induced upregulation of antioxidant defense. Subsequent research revealed that serum SOD1, that responded to varied dietary copper intake similarly to RBC SOD1, was a sensitive indicator of tissue copper levels [10].

Measures of trace element nutritional status accurately predicted the competitive performance of elite swimmers. We derived sex-specific, swim performance models including trace element intakes and blood biochemical measures of trace element status in one sample of elite swimmers [11] then evaluated them in another sample of collegiate swimmers. Actual swim times during national championship competition were not different than predicted swim times with limits of agreement less than 1.5% [12]. These findings established the importance of trace element nutrition in physical performance among athletes within a comprehensive training program.

## Physical stressors to challenge cardiovascular function and energy metabolism

As we considered use of the dual stressor model to studies on the metabolic unit, it became clear that energy homeostasis, demonstrated as constancy of body weight and composition, was needed similarly as providing accurate amounts of trace elements and measuring losses among the study participants. Bolonchuk and I established the first program to assess physical work capacity and body composition then derived individualized exercise prescriptions. This integrated program maintained aerobic capacity, resting metabolic rate, body weight, and composition within 2% of admission values among volunteers living on the metabolic unit up to 12 months [13]. Notably, it eliminated variability in energy balance as a moderating variable in establishing trace element requirements.

Although very low-copper diets had been shown to adversely affect cardiovascular function in rodents, evidence of similar effects in humans consuming marginal copper intakes was not available. We administered a battery of autonomic cardiovascular function tests and included sustained handgrip exercise to healthy women consuming diets containing graded amounts of copper [14]. We found no effect of dietary copper on heart rate, blood pressure or performance of orthostatic maneuvers at rest. Low-copper intake, however, was associated with significantly increased systolic and diastolic blood pressures during the 5-min handgrip test. Copper retention, a classic indicator of copper nutritional status, decreased during consumption of the low-copper diet. Copper-dependent enzymatic ceruloplasmin activity, but not immunoreactive ceruloplasmin, decreased significantly with low-copper diet. The ratio of enzymatic to immunoreactive ceruloplasmin was significantly associated with mean arterial pressure at the end of the handgrip test. These findings revealed a functional defect in human blood pressure regulation during mild copper depletion and identified the ceruloplasmin ratio as a new biomarker of copper status. Sir Roger Bannister, an interested reader of the research report, acknowledged the importance of this finding in a personal note.

Iron deficiency anemia adversely affects energy metabolism during exercise and cold exposure. However, tissue iron depletion without anemia is common in women but its impact on work output and temperature regulation was not known. We addressed this problem in menstruating women residing on the metabolic unit and fed whole-food low iron content diets. Iron depletion was recognized as significantly decreased iron retention and reduced serum ferritin levels. Iron depletion, compared to iron adequacy, resulted in intolerance to cold air in an environmental chamber with an earlier onset of visible shivering, significantly faster rates of body cooling, and decreased metabolic heat production due to impaired non-shivering thermogenesis [15]. Blunted thyroid hormone responses and exaggerated sympathetic nervous system activity explained the impaired thermoregulatory response of the iron-depleted, non-anemic women.

## Exercise as a provocative stressor of physiological function

Exercise is well known to increase demands on many physiological systems. We employed different modes of exercise and identified significant functional deficits associated with marginal intakes of trace elements.

Iron depletion affected energy metabolism during progressive, peak exercise testing. It resulted in significantly increased peak carbon dioxide production, respiratory exchange ratio, and venous lactate concentration [16]. Total oxygen consumption, total aerobic energy expenditure, and the rate of oxygen utilization were significantly reduced due to an increased reliance on glycolytic metabolism after iron depletion, consistent with a previous observation in elite male runners with low serum ferritin levels. The combination of significantly blunted thermoregulatory responses during cold air exposure and reduced aerobic metabolism during progressive exercise emphasize functional impairments in iron-depleted, non-anemic women challenged with some stressors of daily living.

Research in the role of magnesium in physical activity increased following the publication of my initial paper describing a relationship between magnesium status and physical work capacity. Evidence that low dietary magnesium intake was common among physically active men and diet-induced magnesium depletion was rare without concurrent illness encouraged us to investigate the effects of magnesium depletion on biochemical indices of magnesium status and functional responses during exercise in postmenopausal women [17]. Low dietary magnesium intake, consistent with the usual intake of older women resulted in magnesium depletion compared to retention during periods of adequate and supplemental magnesium intakes. Serum magnesium levels remained within the range of normal values with trends of decreases and increases with magnesium restriction and adequacy, respectively. In contrast, RBC and skeletal muscle magnesium concentrations directly and significantly responded to magnesium intake. Dietary magnesium significantly affected metabolic and cardiovascular responses during submaximal exercise. Heart rate and energy cost were significantly greater when magnesium intake was low, compared to adequate or supplemental, with total oxygen consumption inversely and significantly correlated with cumulative magnesium loss. The findings of decreased energy efficiency (increased oxygen needs for the same work) and elevated heart rate during whole body and skeletal muscle magnesium depletion indicate important functional limitations of inadequate magnesium intake. The impaired work metabolism was similar to findings of physically active adults and elite athletes consuming less than recommended magnesium intakes, and provides unique evidence of a common role for magnesium related to the increased energy cost of muscle contraction during exercise.

Zinc is a component of more than 200 metalloenzymes, some of which are involved in energy metabolism during physical activity. Limited evidence suggested that low-zinc intakes can impact physical performance with no evidence of a mechanism. I tested the hypothesis that low dietary zinc intake reduces the activity of RBC carbonic anhydrase, a zinc-dependent enzyme, thus adversely affecting cardiorespiratory function and metabolic responses during exercise in physically active men [18]. Based on reported estimates of zinc intakes of elite athletes, we fed diets low and supplemented with zinc to physically active men in a double-blinded cross-over trial. Serum zinc and RBC zinc concentrations, total carbonic anhydrase activity, and the activities of the carbonic anhydrase I and II isoforms in RBCs decreased significantly when the men consumed the low-zinc diet. When dietary zinc was low, the volunteers with the largest reduction in carbonic anhydrase activity did not complete the 45-min submaximal exercise test. Heart rate, ventilatory volume, and ventilatory equivalents for oxygen and carbon dioxide were significantly increased when the men consumed the low-zinc diet. These findings demonstrate that marginal zinc intake, common among physically active adults, adversely affects cardiorespiratory function and can limit aerobic exercise performance.

During a management training assignment at the National Institute of Diabetes, Digestive and Kidney Disease, Van Hubbard presented me with the advertised claims that supplemental trivalent chromium as chromium picolinate independently caused fat loss and muscle gain. I carried out a double-blind supplementation study using the standard dose of over-the-counter supplements of chromium (picolinate and chloride), compared to placebo, and found no significant effect on either body composition or strength gain in young men after completion of a resistance training program [19]. The Food and Drug Administration reviewed these findings and concluded that the claims of generalized fat loss and muscle accretion with chromium picolinate supplementation were false [20]. A follow-up double-blind study of women fed low-chromium diets and supplemented with chromium (picolinate and chloride) also failed to demonstrate any favorable effects of supplemental chromium, compared to placebo, on body weight or body composition [21].

Publication of these research findings heightened public awareness of the importance of trace elements in health and physical activity, and led to my assignment as the contact scientist for diet and physical activity at the USDA-ARS. The timeliness and importance of my research coincided with program interests at the National Institutes of Health, US Army Research Institute of Environmental Medicine, American Society for Clinical Nutrition, American College of Medicine, and Gatorade Sports Science Institute that organized national and international symposia at which I presented my research. One outcome was my invited comprehensive review of the roles of vitamins and minerals on physical performance for the 2004 Olympic Games [22]; this research continues to be of interest today [23].

Community interest in my research coincided with my participation as a coach of my daughters in various competitive sports. As administrators in sport organizations learned of my research relating nutrition and physical activity, I made presentations at regional and national conferences of scholastic and collegiate coaches and athletic directors. The focus was diet and nutrition for sport, expanded to interpretation of supplement advertisements, and evolved to development of practical guidelines and resources for athletic trainers, and as a contributor to continuing education programs for educators. My role as a translator of scientific information on nutrition, performance, and health advanced when I accepted an invitation from Ron Maughan and joined the faculty of the Diploma Program in Sports Nutrition, International Olympic Committee.

## Renaissance of body composition assessment

After a hiatus of more than 20 y, interest in methods of body composition assessment resumed in response to the emerging obesity epidemic, rising problem of disease-related malnutrition, evolving awareness of sarcopenia, and the clinical need for hydration assessment in patient care. Leadership in the human nutrition program of USDA-ARS tasked me to chair a national symposium and ascertain the status of body composition assessment. At the same time, Nevin Scrimshaw, United Nations University, and Pan American Health Organization invited me to prepare a comprehensive report on body composition assessment methods stressing applications in public health and nutrition research, and to project future needs and opportunities [24]. The principal conclusion was a growing demand to develop innovative, valid, practical, and cost-effective methods for use outside of the laboratory.

## The odyssey of bioelectrical impedance analysis

Bioimpedance analysis (BIA) was known as a non-invasive clinical method to estimate pulsatile blood flow based on the physical principle that water and electrolytes conduct an applied alternating current. My colleagues and I developed and validated the first algorithms based on whole-body bioelectrical resistance measurements using the physical relationship between conductor volume, length, and resistivity to estimate TBW, TBK, and FFM in healthy adults [25–27]. The years following the publication of our paper in 1985 witnessed a rapid acceptance of BIA as demonstrated by the rapid and expansive rise in the number of publications using this method [28, 29]. The popularity of BIA for research and clinical applications was attributed to its practicality, safety, portability, ease of operation, relatively low cost, and the extensive availability of commercial devices. The rapidly growing research interest in BIA resulted in a National Institutes of Health Technology Assessment Conference [30] and a follow-up conference that summarized standardized procedures, current applications, future directions, and cautions for proper uses of this technology [31].

The predominant use of BIA was to quantify body components evolved from whole-body 50 kHz resistance and multiple regression equations to multiple frequency measurements then impedance spectroscopy and biophysical models [32, 33]. Accumulating evidence confirmed cautionary concerns of the limitations and imprecision of these BIA predictive approaches as related to inclusion of body weight as an independent predictor of conductor volume and the assumption of constant hydration of the FFM [34, 35]. These factors stimulated a return to primary BIA measurements as indicators of physiological parameters and emphasized the primary value of their interpretation [36–38].

After retirement from the USDA-ARS, I accepted an adjunct faculty position in the Department of Kinesiology and Public Health Education and extended my pursuit of new applications of BIA in clinical studies that was outside the mission of the agency. Interactions with Antonio Piccoli and Antonio Talluri expanded my understanding of the physiological interpretations of raw BIA measurements, in contrast to use of prediction models to quantify conductor volumes, in clinical applications. Collaborative research resulted in the demonstration of novel uses of BIA including monitoring of wound healing [39], identification of adults at risk of acute mountain sickness based on hydration status [40], and tracking changes in fluid distribution of athletes during training and competition [41].

An important application of raw BIA measurements was localized BIA in traumatic muscle injury to evaluate cellular integrity and fluid distribution. In collaboration with Lexa Nescolarde and colleagues at the Barcelona Football Club, we demonstrated that BIA measurements at the site of the muscle injury were significantly altered compared to the contralateral uninjured leg. All BIA measurements decreased significantly in the region of the injury, and the predominant difference was in reactance that was directly related to the severity of the radiologically-determined muscle injury corresponding to a substantial decrease in membrane capacitance attributed to a reduction in membrane integrity [42]. The lesser effect on resistance indicated an increase in extracellular fluid and shown with a significant decrease in phase angle. Serial localized BIA measurements during recovery revealed increases in reactance and phase angle values comparable to the non-injured muscle site consistent with radiological evidence of muscle injury healing and indicating safe return to play [43].

The widespread use of different BIA devices raised technical concerns about the lack of comparability of BIA measurements. One collaborative study revealed that bioelectrical impedance spectroscopy significantly underestimated resistance and reactance at 50 kHz compared to single-frequency phase-sensitive BIA and concluded that devices are not interchangeable for BIA measurements [44]. Also, differences in the composition of adhesive Ag/AgCl electrodes significantly affected the intrinsic BIA characteristics of the different electrodes resulting in misclassification of hydration status of healthy adults [45]. These findings emphasize the need for standardization of BIA measurement to avoid systemic errors when these measurements are used for clinical interpretations [46].

Phase angle measured at 50 kHz was first recognized as a prognostic indicator of morbidity in the mid-1930’s and gained additional clinical interest 50 y later. Jose Manuel Garcia-Almeida and I assembled a comprehensive series of papers updating the applications of phase angle in health and disease highlighting physical activity, aging, and chronic disease with a discussion of the effects of inflammation and oxidative stress as moderators of phase angle measurements [47]. One consensus was that phase angle is a sensitive biomarker of prognosis: low values indicate poor outcomes. However, definition of cut points or threshold values for prognosis are lacking due to a lack of standardized technical considerations. Importantly, physiological interpretation of a phase angle value requires simultaneous examination of the impedance vector position on the resistance-reactance plot in bioelectrical impedance vector analysis [48]. This requirement for concomitant evaluation of hydration and cell mass is particularly important in detecting the nutritional status of a patient in the presence of altered fluid status [49, 50].

A second retirement project was to assemble updated comprehensive reviews that critically evaluated the role of body composition assessment in health and physical activity. I edited a book that filled the knowledge gaps in understanding the roles of muscle, fat, and bone in facilitating physical performance and health in sports and physically demanding occupations [51]. An invited review highlighted functional body composition assessment to benefit health, prevent injury and optimize performance in sport [52].

## Crystal ball thoughts

Clinical nutrition is an amalgamative science that can contribute to the prevention of non-communicable chronic disease (NCCD) and enhance the quality of life of a population. It draws from various scientific disciplines to integrate findings from different experimental models to improve health and optimize function. It utilizes new technologies and methods to determine mechanisms of dietary factors in health promotion. Attention to the modifiable factors of diet and physical activity is a current focus to attenuate the adverse effects of obesity on morbidity and mortality and to promote the preservation of muscle mass to maintain physical function in an aging population. Progress in these areas requires participation of non-traditional partners who can improve awareness of the value of healthy diets and the importance of considering cultural and social diversity, sustainable agricultural practices, economics, behavioral and mental health as barriers in development of public health nutrition programs. Teams of cooperating researchers, therefore, are needed to address the breadth of complex issues of health promotion and formulate solution-based research outcomes.

The concept of “team” is fundamental in clinical nutrition and consistent with the message of Aristotle that the “whole is greater than the sum of its parts.” My wife and daughters provided unwavering support, patience, and love, and constituted my “home team.” My “professional team” consisted of colleagues at the GFHNRC, who generously shared technical knowledge and expertise, and leadership at the USDA-ARS that utilized my input to develop and implement national nutrition research programs. Colleagues at other governmental agencies, academic institutions, and professional organizations engaged in productive interactions and provided occasional funding for collaborative research. The team approach has exceptional value as it enhances communication to share ideas, identify gaps in knowledge, and foster creativity among individuals with diverse scientific backgrounds and knowledge with a common interest.

A corollary of the “team” approach is mentorship. As a research leader in the USDA-ARS, I welcomed high school and undergraduate students from underserved groups for introductory experiences in science. The program was attractive to the students because the hands-on experiences involved projects in basic nutrition, body composition assessment, and exercise physiology that were relevant to their personal interests. Outcomes were largely positive with some students achieving recognition at regional science competitions and many others completing undergraduate programs in health-related programs and medical school. Similar positive outcomes occurred with post-doctoral research associates, who advanced to academic or research positions, and visiting scientists. These experiences emphasize the importance of extensive team building for professional development of early career scientists for future advancement of clinical nutrition.

The horizon of clinical nutrition research reveals exciting opportunities. Assessment of nutritional risk at admission to hospital and among critically ill patients remains a challenge. Recent findings indicate the value of phase angle as an index of altered nutritional status based on depletion of body cell mass. Inclusion of fluid status classification with cell mass assessment has the potential to improve determination of disease-related malnutrition. An emerging method is morphofunctional assessment that includes detection of body cell mass or FFM depletion with concurrent assessment of physical and mental function in response to starvation, disease, or aging [53]. Each of these methods has potential value in identification of nutritional deficits and evaluation of interventions in disease-related malnutrition and sarcopenia.

Pressing societal demands to enhance the quality of life and to reduce morbidity and mortality from chronic disease will continue to rely on factors controlled largely by individuals. Clinical nutrition offers a bridge from the laboratory bench to individuals in communities to meet this need. Scientists, either by focused training or “accident,” are needed to contribute to this mission.
